# Control of visible-range transmission and reflection haze by varying pattern size, shape and depth in flexible metasurfaces

**DOI:** 10.1007/s12200-024-00125-3

**Published:** 2024-07-30

**Authors:** Avijit Maity, Vaswati Biswas, R. Vijaya

**Affiliations:** 1 Department of Physics, Indian Institute of Technology Kanpur, Kanpur, 208016 India; 2 Centre for Lasers and Photonics, Indian Institute of Technology Kanpur, Kanpur, 208016 India

**Keywords:** Metasurface, Reflection haze, Transmission haze, Soft imprint lithography

## Abstract

Cost-effective soft imprint lithography technique is used to prepare flexible thin polymeric surfaces containing a periodic arrangement of nanodimples and nanobumps of sub-micron size. Using a single master mold of self-assembled colloidal crystal, metasurfaces with different depths and heights of patterns with a fixed pitch are possible, which makes the process inexpensive and simple. These metasurfaces are studied for their diffuse and total transmission and reflection spectra in the visible range. The transmission haze and reflection haze are calculated from the measurements. The surface containing nanobumps of lesser pattern height result in higher values of reflection and transmission haze than from surfaces containing nanodimples of much higher depth for the same pitch. The haze is more dependent on the pattern depth or height and less dependent on the pitch of the pattern. Far-field transmission profiles measured in the same wavelength range from the patterned surfaces show that the scattering increases with the increase of the ratio of pattern depth/height to pitch, similar to the haze measurements conducted with a closed integrating sphere. These profiles show that the angular spread of scattered light in transmission is within 10°, explaining the reason for the relatively low transmission haze in all the patterned surfaces. Simulation results confirm that the nanobump pattern gives higher transmission haze compared to nanodimple pattern. By controlling the ratio of pattern depth/height to pitch of the features on these surfaces, both an increase in optical haze and a balance between total reflection intensity and total transmission intensity can be achieved.

**Graphical Abstract:**

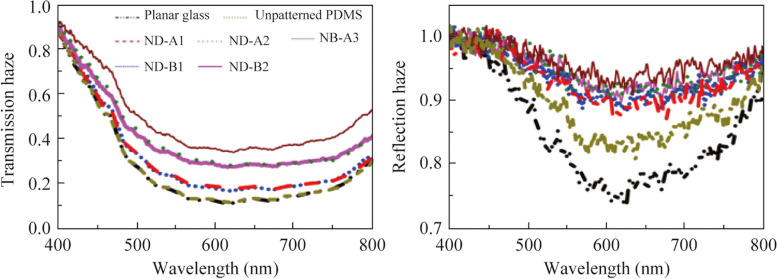

## Introduction

Nanopatterned surfaces have been studied for more than a decade for their effectiveness in controlling electromagnetic waves through interaction with artificial meta-atoms present on their surface [1–3]. These meta-atoms act as scattering elements exhibiting exotic electromagnetic responses, such as ultra-high index or negative index or zero index. This is due to their specially-designed shapes arranged either periodically or randomly on the surface [4]. Such surfaces are more effective in controlling the properties of electromagnetic waves than planar surfaces, and make it feasible to realize novel effects in the form of wavefront manipulation, wavefront shaping [5, 6], polarization conversion [7, 8] and radiation control [9, 10]. Pattern sizes in the sub-wavelength scale have the capacity of strong wavelength control enabling many applications such as meta-lens [11–14], invisible cloak [15], absorber [16–18], vortex beam generator [19], and in holography [20–22], making patterned surfaces useful for optoelectronic applications. Characteristics associated with polarization, phase, propagation direction and amplitude of electromagnetic waves can be controlled using nanopatterned surfaces, opening the possibility for color management, sensing and biomedical applications [23–25].

Since the optical properties of reflection, transmission, absorption and scattering depend on the pattern size, depth/height of the pattern, spacing between the patterns and the wavelength of incident electromagnetic waves, apart from the refractive indices of the pattern material and the substrate material, nanopatterned surfaces display more interesting phenomena than uniform homogeneous surfaces [26, 27]. For improvement in the performance of optoelectronic devices, it is important to control light propagation characteristics inside the thickness of the material layer. In recent years, nanopatterned surfaces on flexible or hard layers have improved the efficiency of many optoelectronic devices such as the light emitting diodes (LED), smart windows, and solar cells [28–30]. Devices which work on the principle of absorption of electromagnetic energy tend to show poor efficiency in thin layers due to a single passage between smooth interfaces. A large portion of the electromagnetic energy that remains unused in such cases can be made use of, and the absorption efficiency improved, using nanopatterned surfaces present at the interfaces. This can be useful to minimize the reflection loss or provide multiple paths in transmission, thus increasing the extent of absorption [27, 29]. Nanostructured layers are effective in increasing light absorption in a broad spectral range and can act as anti-reflection (AR) surfaces too [29–31]. It is often expected that a reduction of *specular* reflection at an interface implicitly increases transmission. In this context, it is important to differentiate between *specular* and *total* reflection, as well as *specular* and *total* transmission. Any presence of *diffuse* reflection and *diffuse* transmission can influence the balance between specular and total reflection/transmission. Thus, a reduction of *specular* reflection at an interface may not increase *specular* transmission. This is especially a relevant case for nanopatterned surfaces, where diffuse effects in reflection and transmission can increase, leading to haze in specific wavelength ranges.

Study of optical haze is important for several optoelectronics applications. More transmission haze at a surface or interface is good for applications such as photovoltaic cells and photodetectors which depend merely on the optical to electrical conversion efficiency [3, 32, 33]. Surfaces that are free of reflection and transmission haze are useful in applications related to smart windows, touch-panels, see-through displays, and ranging studies [2]. In many optoelectronic devices, silicon (Si) is the primary material. But it has high reflection (higher than 30%) and correspondingly lower transmission at air-Si interface due to its high refractive index (~3.4) in the visible range of wavelengths. Its reflection, transmission and haze can be controlled using different techniques. Oh et al. have reported super-hydrophobic surface using a scalable spraying process with silica nanoparticles, which is haze-free and applicable in smart windows, touch-panels, and see-through displays [2]. Marus et al. showed that optical haze can be controlled using randomly arranged silver nanowire transparent conductive films. They showed that optical haze increases by 8 times when thickness of the nanowires increases from 30 to 100 nm [32]. Choi et al. reported that using MgF_2_ film coated SU8 micro-cones on Si substrates, average reflectance reduced compared to the bare Si and acted as anti-reflective surface. In addition, the MgF_2_/SU8 micro-cones showed a high transmission haze without affecting the total transmission [33]. Light capture, light haze and transmission can be improved by the surface modification of silicon, inspired by leaf epidermis structures [34]. It is also reported that artificial inverted compound eye structure with hierarchical nano-textures/periodic micro-grating can increase the haze and transmittance compared to the bare glass [3]. Even a random size inverted-pyramid structure polydimethylsiloxane (PDMS) sticker on bare glass and c-Si substrate reduces the reflectance by a significant value [31].

The methods and techniques discussed above for controlling haze have used different processes, materials and patterns. Some of them involve complex designs or they are difficult to fabricate or they require a high cost of production. Some have drawbacks in aspects such as durability, thermal expansion mismatch, and poor or reduced adhesion on certain substrates [33]. There is no single method which is well-suited for controlling all the properties of light over a wide spectral range and for all angles of incidence. This has pushed the studies more toward biomimetic designs, inspired by artificial compound-eye structures present on the corneas of insects which act as graded-index surfaces that reduce surface reflection and enhance the transmission [29, 33]. Nanopatterned surfaces which replicate moth-eye arrangement give reflection independent of angle or wavelength of the incident light [27, 29, 30]. They can also minimize total internal reflection losses in optoelectronic devices. Moth-eye patterning with soft lithography on PDMS is cost-effective and scalable in comparison to the energy-intensive mechanized patterning methods such as electron-beam lithography, photolithography, and hard nanoimprint lithography, which require expensive tools. PDMS layer can also easily stick to the substrates that require patterning due to its strong adhesion property. In addition, it is lightweight too, while possessing some essential properties such as transparency, flexibility, chemical/mechanical stability, and biocompatibility [29–31, 33].

When light is incident on the surface of a non-absorbing material, some part of it gets reflected and some part gets transmitted as both specular and diffuse light. It is known that smooth surfaces have more specular and less diffuse components in reflected and transmitted light. Patterned surfaces can result in more diffuse components in both the forward and backward directions. But the relative contribution to haze in forward (transmission) and backward (reflection) directions and its wavelength dependence has not been studied extensively as it depends on the pattern size, shape, placement, index modulation within the layer, and the dielectric contrast with the substrate. Some optoelectronics applications (such as the solar cell) require high transmission haze into the active layer but low reflection haze into air, while some others (such as the LED) may require very high reflection haze into the active layer to increase the eventual extraction efficiency into air, along with preferred wavelength effects. On the other hand, smart windows will require both reflection and transmission haze to be low, while personal devices such as smartphone or laptop screens will require directional (haze-free) transmission to the intended user while having high reflection haze of ambient light for the non-users in the neighborhood. Hence, it is an on-going research effort to identify surfaces that enhance or reduce haze to a specific extent.

In this work, we have studied both transmission haze and reflection haze, along with their wavelength dependence, from inexpensive polymeric metasurfaces. The biggest advantage is the systematic variation in haze possible from these metasurfaces. We made self-assembled photonic crystals in-house, and used soft lithography technique to prepare the patterned surfaces in PDMS using the photonic crystals as master molds. It is therefore doubly economical due to inexpensive master mold and simple patterning process. The versatility is further increased as a variety of pattern depths are possible from a single master mold, and the same master mold can be re-used for multiple patterning cycles. In addition, self-assembled photonic crystals can be grown with colloids of different diameters, providing patterned surfaces with different values of pitch. With the help of double replication in soft lithography, metasurfaces were made with opposite curvatures of unit cells. This dual choice of pattern shape from the same master mold provides the advantage of two different graded refractive index profiles within the thickness of the metasurface. With the help of such a comprehensive set of patterned surfaces of PDMS, we demonstrate that we can increase the diffuse reflection (and reflection haze) and diffuse transmission (and transmission haze) over the entire visible wavelength range, without much effect on the total reflection or total transmission. The study of wavelength-integrated far-field transmission establishes minimal spread of transmitted light from the patterned surfaces. This is useful in increasing the efficiency in optoelectronic devices where both high values of total transmission and diffuse transmission or haze are required. Moreover, the patterns on these metasurfaces increase the reflection haze by increasing the diffuse reflection even though specular reflection gets lowered by the patterning. Thus, these metasurfaces are ideal for applications where the presence of an additional surface does not lead to specular reflection loss, but improves the reflection and transmission haze to the required extent. Simulation results on a set of nanodimple and nanobump metasurfaces with comparable pitch establish that the nanobump metasurface gives higher transmission haze even when its pattern height is lesser than the pattern depth of the nanodimple metasurface.

## Description of samples, their fabrication technique and structural characterization

Planar glass and unpatterned PDMS layer are used as reference samples for the optical studies of transmission and reflection, since their reflection and transmission spectra in the visible range are predictable from their refractive index values. In addition, thin layers of PDMS patterned with different values of pitch and depth/height have been studied in this work. The pattern shapes are either dimples or bumps on the surface of PDMS. All the dimples and bumps of different pitch/depth/height have shapes that are a part of a sphere and are always arranged in a triangular lattice, as they are replicated from the top surface of self-assembled photonic crystals grown using colloidal spheres. All the patterned PDMS layers are laminated on glass during the measurements for the ease of handling. But they can be used independently in their flexible condition for any conformal measurements if required.

Metasurfaces with pattern sizes in the range of hundreds of nm are required for applications in the visible range of wavelengths. Patterning such small sizes uniformly on a surface is difficult and requires special methods. One approach is to use high-precision mechanized methods such as electron beam lithography or focused ion beam milling to directly create the patterns on a surface. These methods are capable of providing extremely precise patterns with very small pitch, and with highly identical pattern shapes. But they are slow processes, require expensive equipment, work only with certain photoresists or substrates, and are suitable for small areas of the order of a few μm^2^. Another approach is to use replication methods using a master mold created using one of these methods on a hard surface, and replicating the pattern multiple times using an elastomeric material. Metasurfaces meant for optoelectronic applications require patterned areas that are bigger in size (of the order of optical beam area in mm^2^) and that are suitable to the device under study. The cost, the manufacturing time and the material of the metasurface are also factors that influence the utility.

In this work, we used a low-cost three-dimensional colloidal photonic crystal (3D-PhC) as a master mold to prepare the metasurfaces. This avoids the use of an expensive commercial master pattern. The 3D-PhC was fabricated using the inward growing self-assembly method [35] over a large surface area of a few cm^2^ using aqueous suspensions of monodispersed polystyrene colloids (Microparticles GmbH, Germany). High quality 3D-PhC were grown from different colloidal sizes without any specialized equipment, within a duration of a few hours in this method. The values of average colloidal diameters were 196, 277, 557, and 757 nm in this study. In the 3D-PhC, the self-assembly process ensures that the colloids are arranged in a triangular pattern within each layer and are also stacked in multiple layers, with a close-packed face-centered cubic lattice arrangement among the layers. The replication process uses only the top-most layer of the 3D-PhC and hence the periodic arrangement is fixed as a triangular lattice. After fabricating the 3D-PhC and drying it completely, the replica of its top surface was taken on PDMS polymer. To do this, the PDMS solution with predetermined ratio of base to curing agent was cast on the master and the thin elastomeric layer of PDMS was peeled off after it is solidified [29, 30, 36]. This first replication step yields a non-close packed air-filled 2D nanodimple array present in a triangular arrangement. The nanobump metasurface was fabricated using a second step of replication process, utilizing the nanodimple metasurface as a master [37, 38]. In our work, nanodimple metasurfaces along with nanobump metasurfaces were employed to investigate the haze effect in both reflection and transmission geometry.

In this study, metasurfaces with various values of pitch as well as different pattern depths and height were used. The average diameter of the colloids in the 3D-PhC master decided the pitch of the metasurfaces, while the viscosity of the PDMS polymer employed in the replication processes determined the pattern depth and height. Smaller viscosity of the PDMS polymer yields larger depth (height) of the patterns in the nanodimple (nanobump) metasurface [30]. The maximum depth feasible for the pattern in a nanodimple metasurface is equal to the radius of the colloids used to fabricate the 3D-PhC, with the dimples in contact with one another, while shallower depths result in dimples that are far apart from one another. Similarly, the maximum possible height for the patterns in nanobump metasurface is equal to the radius of the colloids; however, because the nanobump metasurface was fabricated after a second replication using a nanodimple metasurface as a master, its pattern height is decided only by the depth of the patterns in the nanodimple metasurface used as the master. As a result, we have shallower heights of the bumps in a non-close packed arrangement in a triangular design in the nanobump metasurface.

**Fig. 1:**
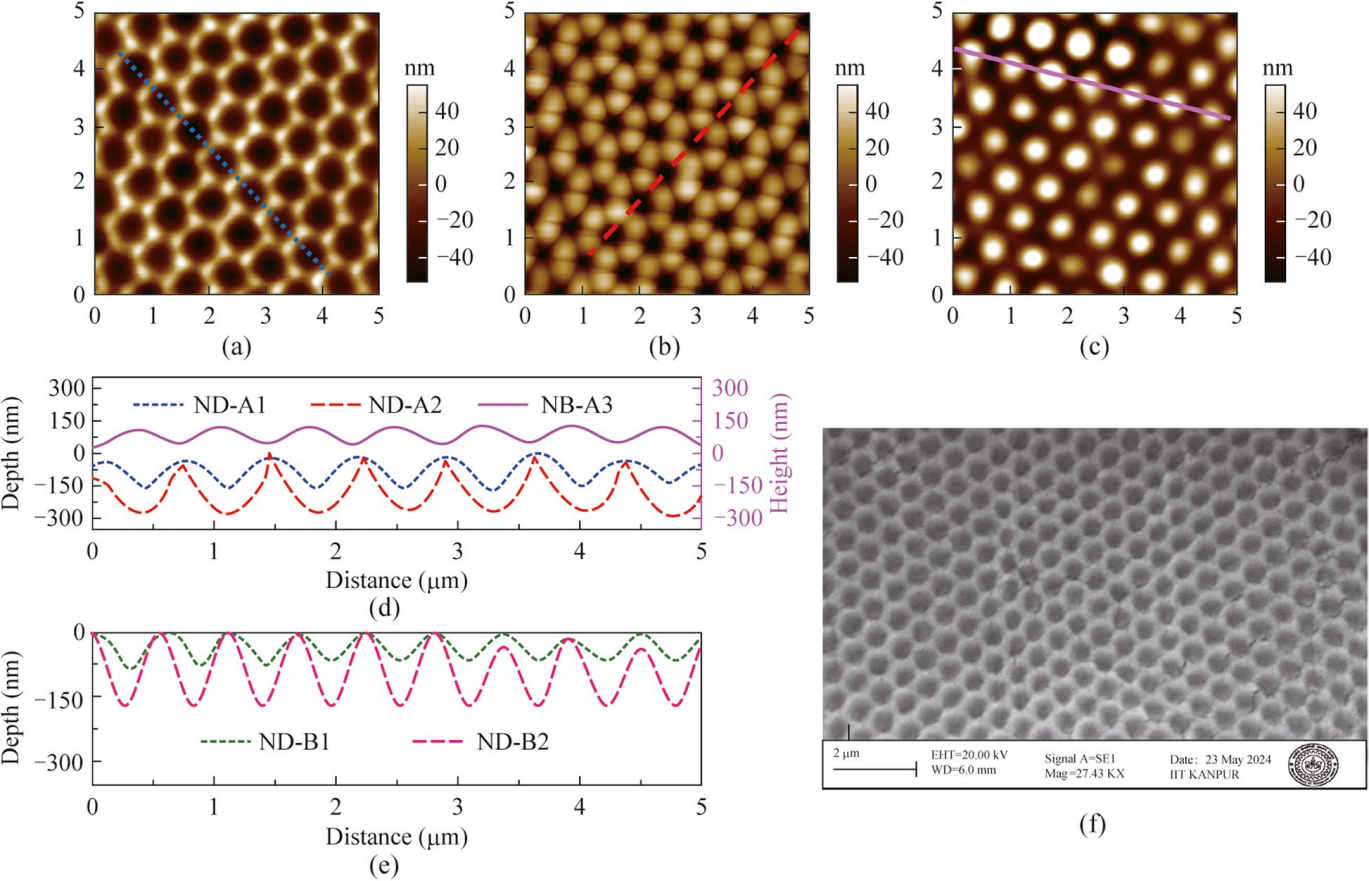
AFM images of the top view of ND-A1, ND-A2 and NB-A3 are shown in **a**, **b** and **c** respectively. The line profile plots of **a**, **b**, **c** are shown together in **d**. The lines drawn on the AFM images indicate the position of line profile data. Line profile plots of another set of samples named ND-B1 and ND-B2 are shown in **e** and their AFM images are not shown here. In **f**, the SEM image of the top surface of ND-A2 is shown. The scale bar is 2 μm

The values of pitch and pattern depth/height were obtained from the images shown in Fig. 1 recorded with the atomic force microscope (AFM) (Asylum Research; Oxford Instruments). Figure 1a and b show the AFM images of two different nanodimple metasurfaces (ND-A1 and ND-A2) made from the same 3D-PhC master mold, while Fig. 1c shows the AFM image of nanobump metasurface (NB-A3) made using a nanodimple metasurface as the master mold. Figure 1d depicts the line profiles obtained from these three AFM images along the lines drawn on the images in Fig. 1a−c. The depth profiles of the nanodimple metasurfaces are shown with negative values (*y*-axis on the left) in Fig. 1d, while the height profile of the nanobump metasurface is shown with positive values (*y*-axis on the right). The line profile plots of another set of nanodimple metasurfaces (ND-B1 and ND-B2) made using a 3D-PhC containing smaller colloids are shown in Fig. 1e and these are obtained from their respective AFM images (not shown here). These line profiles are used to get the average values of pitch and depth (height) of the patterns. The scanning electron microscopy (SEM) image of the top surface of a nanodimple metasurface (ND-A2) is shown in Fig. 1f. It confirms the shape and placement of the patterns in the nanodimple metasurface.

The complete set of average values of pitch and depth (height) of the metasurfaces studied in this work are listed in Table 1. Apart from the characterizations shown in Fig. 1, there are two more nanodimple metasurfaces (ND-C and ND-D) of smaller pitch which are used for comparison in the section on wavelength-integrated far-field transmission studies. The average colloidal diameter given in Table 1 is from the manufacturer’s datasheet. The values of average pitch do not exactly equal the average colloidal diameters used in growing the 3D-PhC master mold. Such differences are due to the size distribution of 5% in the monodispersed colloids used in fabricating the 3D-PhC master and the minor variations that occur when the PDMS layer is peeled off from the master. It is clear from Table 1 that the set of nanodimple metasurfaces provide a wide range of pattern depths. In addition, there is one nanobump metasurface (NB-A3) whose pitch is comparable to two nanodimple metasurfaces (ND-A1 and ND-A2) and thus is useful for analyzing the role of pattern shape.

**Table 1:** Tabulation of average pitch, average depth (height), and average normalized depth (height) values of all the samples studied in this work

Sample	Average colloidal size in 3D-PhC (nm)	Average pitch (*p*) (nm)	Average depth (*d*) (nm)	Average height (*h*) (nm)	Normalized depth (*d*_n_)	Normalized height (*h*_n_)
ND-A1	757	715	128.0	–	0.179	–
ND-A2	757	730	248.6	–	0.340	–
NB-A3	757	716	–	72.1	–	0.100
ND-B1	557	566	71.0	–	0.125	–
ND-B2	557	561	172.7	–	0.307	–
ND-C	277	255	14.0	–	0.054	–
ND-D	196	198	7.0	–	0.035	–

As the samples of different values of pitch and multiple values of depth or height have to be analyzed, we define two dimensionless quantities known as normalized depth (*d*_n_) and normalized height (*h*_n_). These are obtained as

1 $${d}_{\text{n}}=\frac{d}{p},$$

2 $${h}_{\text{n}}=\frac{h}{p},$$

where *d* is the average depth of the nanodimple patterns, *h* is the average height of the nanobump patterns and *p* is the feature spacing (or pitch) of the patterns on a chosen metasurface. The normalized depth and normalized height can have values from 0 to 0.5 due to the method of fabrication of the patterned surfaces used in this work.

**Fig. 2:**
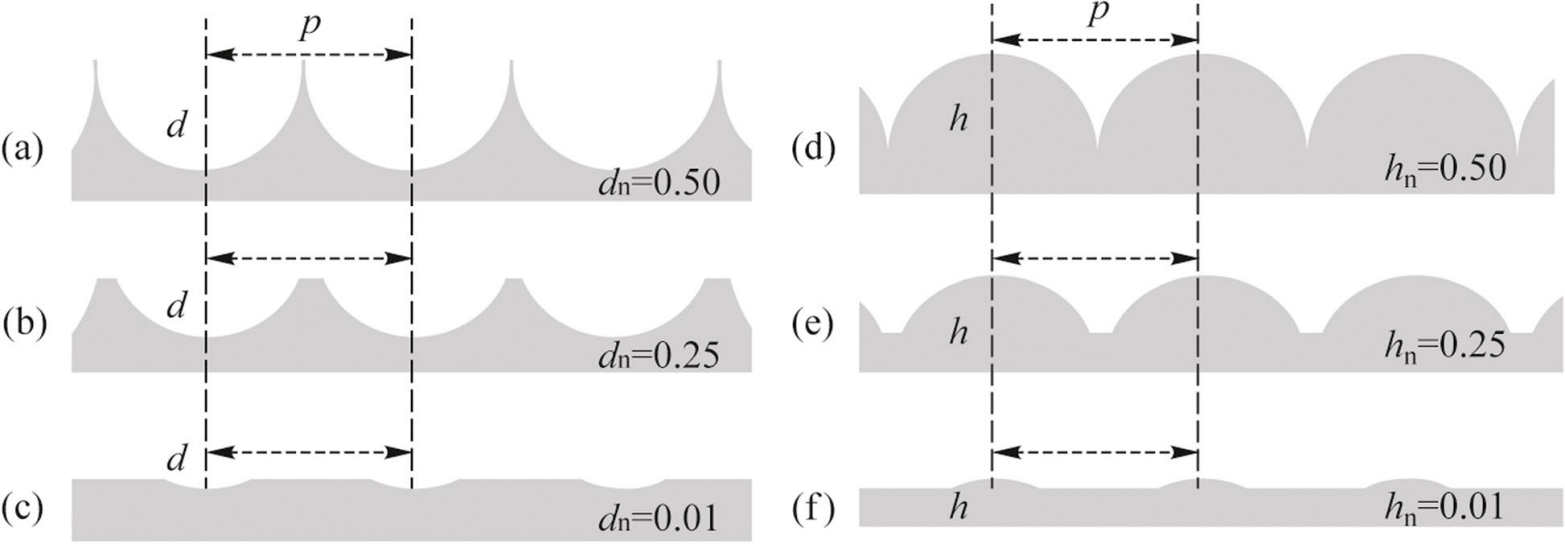
Schematic to demonstrate that nanodimple metasurfaces of constant pitch but different depth of the pattern yield different values of normalized depth *d*_n_ in **a**, **b** and **c**, while nanobump metasurfaces of constant pitch and different height of the pattern lead to different values of normalized height *h*_n_ shown in **d**, **e** and **f**

Figure 2a−c depict the schematic of the variation of *d*_n_ with the depth of the patterns. Figure 2a is for the case of nanodimples with *d* = *p*/2, so that *d*_n_ = 0.50, while Fig. 2b, c show the cases of *d*_n_ = 0.25 and 0.01 respectively. Similarly, Fig. 2d, e, and f represent nanobump surfaces which have *h*_n_ = 0.50, 0.25, and 0.01, respectively. As mentioned earlier, the shape of the curvature of the patterns will be a part of a sphere in all our patterned samples. The values of *d*_n_ or *h*_n_ for all the samples studied in this work are also listed in Table 1. Apart from planar glass and unpatterned PDMS, we have studied nanodimple metasurfaces with *d*_n_ = 0.179 and 0.340 for *p* = 715 and 730 nm (ND-A1 and ND-A2), and *d*_n_ = 0.125 and 0.307 for *p* = 566 and 561 nm (ND-B1 and ND-B2). We have studied one nanobump metasurface with *h*_n_ = 0.100 and *p* = 716 nm. In addition to the above, two more nanodimple surfaces with smaller values of *d*_n_ = 0.054 and 0.035 having *p* = 255 and 198 nm respectively are also used in this study.

## Measurement and analysis of haze

The measurement of wavelength-dependent haze requires multiple quantities to be measured and compared. For the case of transmission haze, these are diffuse transmission intensity and total transmission intensity, both measured as a function of wavelength from the samples. In the case of reflection haze, these are the spectral measurements of diffuse reflection intensity and total reflection intensity. The spectral profile of the source spectrum should be removed from these quantities and the independent contribution of the sample has to be extracted. Hence, measurements are also made for these quantities in the absence of the sample. Diffuse and total intensities are measured using a detector connected to an integrating sphere, with the suitable choice of open/closed ports on the sphere. There are two possibilities of transmission and reflection mode for measurement. Initially, two different wavelength-dependent ratios are analyzed. These are diffuse transmission (reflection) and total transmission (reflection) when the mode of measurement is transmission (reflection). We define these as

3 $$\text{Diffuse transmission}/\text{reflection}=\frac{\text{Diffuse intensity counts with sample}}{\text{Diffuse intensity counts without sample}},$$

4 $$\text{Total transmission}/\text{reflection}=\frac{\text{Total intensity counts with sample}}{\text{Total intensity counts without sample}}.$$

These ratios help in removing the spectral dependence of the source intensity and are also dimensionless. As scattering in the visible wavelength range increases in the presence of nano-patterned samples, the diffuse transmission (and reflection) as defined above can have values more than unity. On the other hand, total intensity is the sum of specular and diffuse intensity. This value in the presence of the sample cannot exceed the incident intensity measured in the absence of the sample. Therefore, total transmission (and reflection) can have a maximum value of unity. From the above spectral measurements, the numerical value of haze can be found using Eq. (5) [33].

5 $$\text{Haze}=\frac{\text{Diffuse intensity counts with sample }}{\text{Total intensity counts with sample}}.$$

Haze is dimensionless and its value will lie in the range of 0 to 1. It can be calculated for transmission and reflection independently based on the mode of measurement. In the following paragraphs, we will discuss the results of both modes of measurement on the planar and patterned samples listed earlier.

### Measurement and analysis of transmission haze

**Fig. 3:**
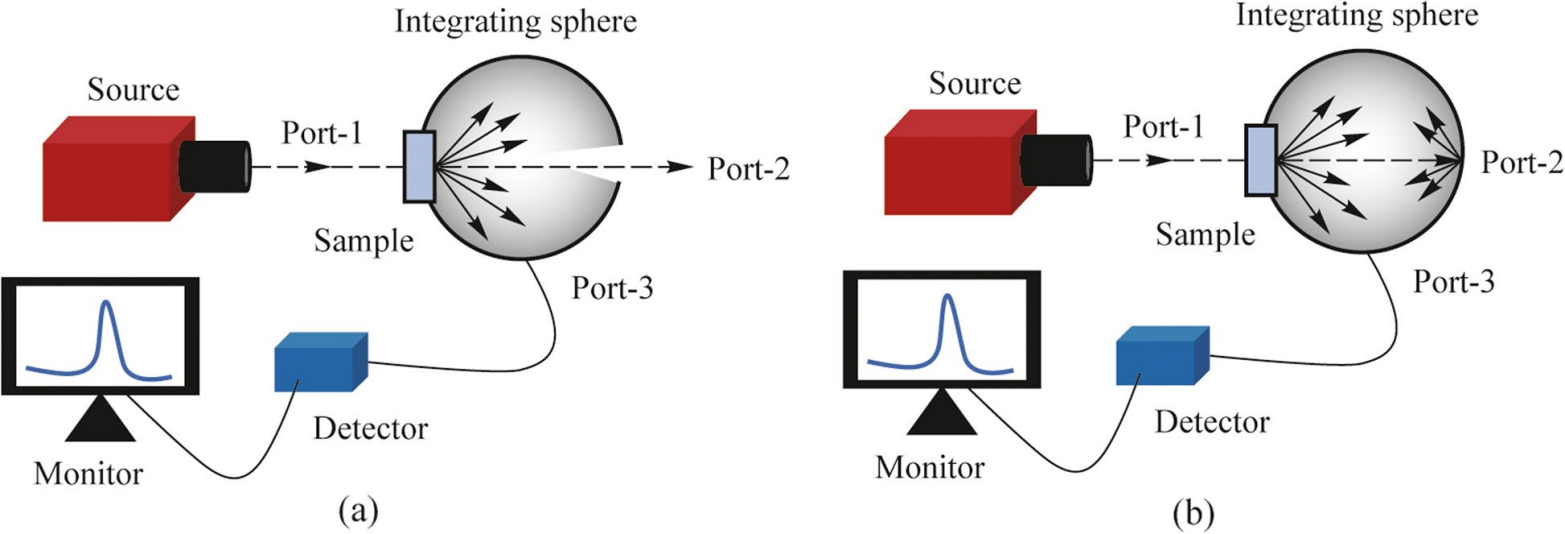
Schematic of the experimental arrangement used to measure diffuse transmission and total transmission is shown in **a** and **b**, respectively

The analysis requires the measurement of diffuse transmission from the samples as well as total transmission, both as a function of wavelength. Therefore, the experimental arrangement consists of a white light source, an integrating sphere which is required for diffuse and total intensity measurements, and a wavelength-selective measurement system. Figure 3a shows the arrangement used for measuring the diffuse transmission and Fig. 3b is the arrangement used for measuring the total transmission.

**Fig. 4:**
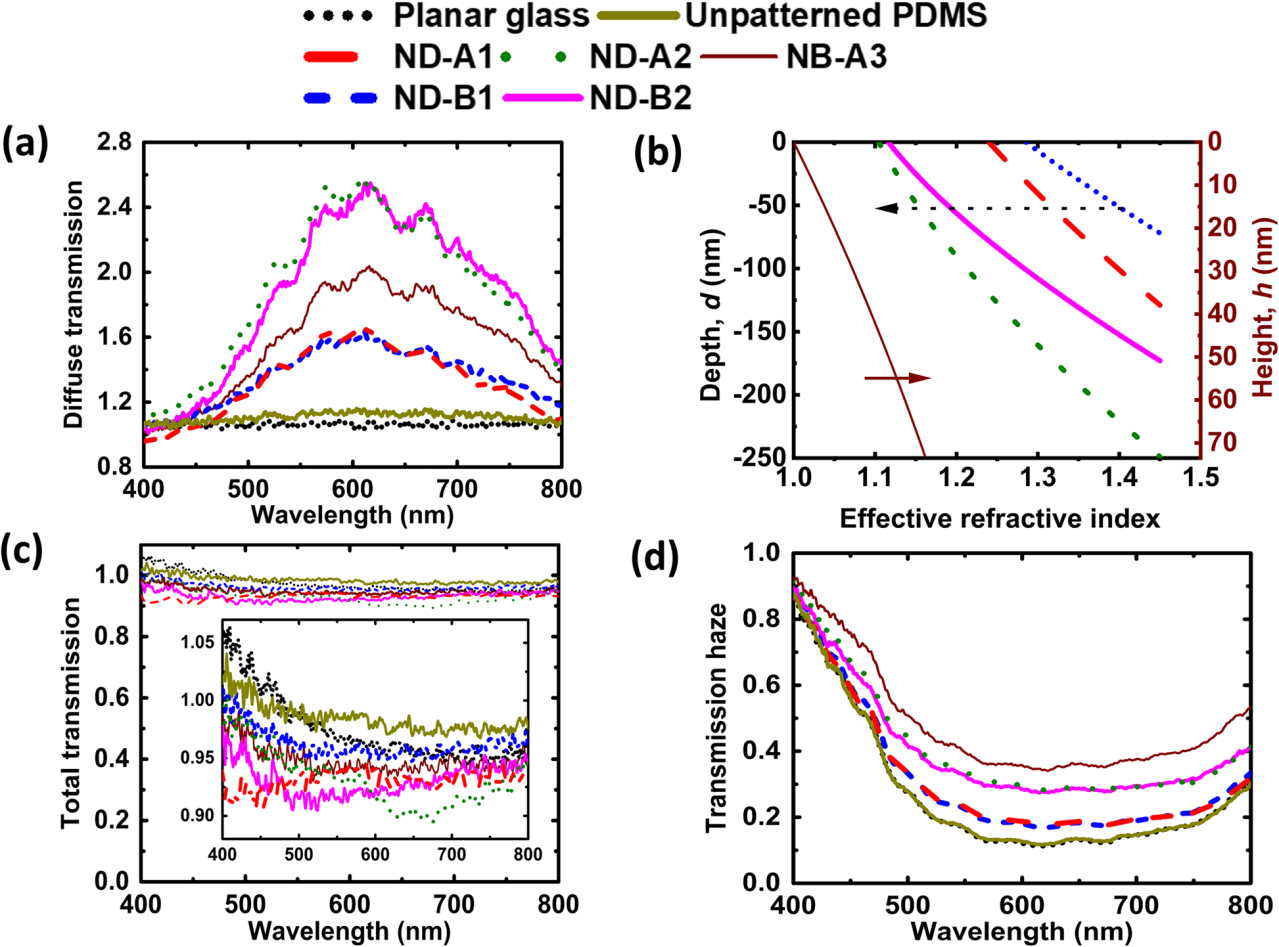
**a** Diffuse transmission. **b** Effective refractive index calculated at different depth/ height of the features. The arrows indicate the relevant y-axis for the data. **c** Total transmission. Zoomed plot in a smaller y-axis range is shown as inset. **d** Transmission haze as a function of wavelength in the visible range

A white light source (Thorlabs, SLS201L) that emits in the wavelength range of 337 to 1760 nm is used as the source. A fiber-coupled detector connected to a spectrometer (StellarNet, detector module for visible) is used for recording the optical intensity (in counts). The integrating sphere (Labsphere, 5.3-inch diameter, spectralon coated) is useful to measure both the diffuse and total transmission by keeping the sample at its entrance port (Port-1), and blocking or opening suitable ports as required in the measurement. Three ports of the sphere are used, wherein Port-1 and Port-2 are 180° apart in the horizontal plane, and Port-3 is at the bottom of the sphere. The source is kept at such a distance from the integrating sphere so that sufficient intensity of light is normally incident on the sample. For these measurements, the samples are kept just outside of the sphere at Port-1 to reduce the effect of any reflected light contributing to transmission counts. During both the diffuse and total transmission measurements, the fiber ferrule collecting the light for the detector is threaded into Port-3. Moreover, Port-2 is kept open while measuring the diffuse transmission in order to remove the specular transmission part contaminating the measurement, while Port-2 is kept closed during the total transmission measurement to collect both the diffuse and specular transmissions. Diffuse transmission and total transmission are measured in the visible wavelength range of 400 to 800 nm from planar glass, unpatterned PDMS and patterned PDMS samples (containing nanodimples or nanobumps of different sizes and depths/heights). Diffuse transmitted intensity (in counts) is measured initially without placing the sample, using the schematic shown in Fig. 3a. This gives the spectral dependence of the light source. Then, the diffuse transmitted intensity is measured by placing the sample in Fig. 3a. The ratio of these two values given by Eq. (3) is plotted in Fig. 4a for various samples studied in this work. This set of samples provide a comprehensive understanding for the role of feature spacing and normalized depth in the optical properties.

In Fig. 4a, planar glass has the lowest diffuse transmission ratio and a value close to 1. This is expected because the glass is highly transparent in the specular transmission direction in the visible range of wavelengths. Unpatterned PDMS is also found to have very low diffuse transmission but its value is slightly higher in Fig. 4a compared to planar glass because PDMS is present on the glass substrate during this measurement. The wavelength dependence is insignificant for both the planar samples. ND-B1 with *d*_n_ value of 0.125 has higher diffuse transmission, while ND-B2 with larger *d*_n_ ( = 0.307) has even higher diffuse transmission. The value exceeding 1 and ranging till 2.5 indicates that the diffuse intensity with sample is more than the value without the sample in transmission mode. ND-A1 sample with *d*_n_ = 0.179 and ND-B1 sample with *d*_n_ = 0.125 have comparable values of diffuse transmission throughout the wavelength range. Similarly, dimpled surfaces with larger pattern depth (ND-A2 with *d*_n_ = 0.340 and ND-B2 with *d*_n_ = 0.307) have comparable but much higher values of diffuse transmission than the dimpled surfaces with lesser pattern depth. The diffuse transmission increases with increase of wavelength up to 600 nm and decreases thereafter for the patterned samples. The wavelength dependence seen in patterned surfaces is clearly due to the surface characteristics of the samples. Depending on the pattern pitch and depth/height, a particular wavelength range shows more scattering compared to other wavelength ranges. As the pitch in the samples have values in from 550 to 750 nm in this discussion, and the wavelength range used is 400 to 800 nm, more diffuse transmission is observed in the range of 550 to 750 nm. The variation of surface profile with normalized depth/height has influence on the wavelength dependence of the analyzed quantity in the patterned samples. This is because with the increase of normalized depth/height, the ratio of patterned to flat region increases, leading to more diffused light. This variation of diffused light with surface profile has been discussed in a latter section.

Nanobump surface (NB-A3 with *h*_n_ = 0.100) has an intermediate value of diffuse transmission lying between the two cases of nanodimpled surfaces of ND-A1 with *d*_n_ = 0.179 and ND-A2 with *d*_n_ = 0.340. One may note that the nanobump surface (NB-A3) has a pitch of 716 nm and the lowest value for the normalized parameter (*h*_n_ = 0.100) among all the patterned surfaces. Relatively higher diffuse transmission seen in the nanobump surface may be attributed to the difference in graded index profile seen between the three surfaces with pitch more than 700 nm as shown in Fig. 4b. The graded index profile, i.e., the variation of effective refractive index with depth, *d* (for nanodimples) or height, *h* (for nanobumps), is calculated by considering the 2D hexagonal lattice unit cell with circular disk containing air for nanodimples and containing PDMS for nanobump. Each dimple or bump in the unit cell is sliced into layers of 1 nm thickness (*t*) and fill fraction of PDMS (and air) is calculated for the unit cell for each layer in the depth/height by considering the shape of dimples/bumps as a part of a hemisphere. Using fill fraction at each layer, effective refractive index can be calculated at different depth or height using [37, 39]:

6 $${n}_\text{eff}\left(t\right)=\sqrt{{{n}^{2}}_\text{pdms} f\left(t\right)+ {{n}^{2}}_\text{air} (1 -f\left(t\right))},$$

where *f*(*t*) is the fill fraction of PDMS at a depth *t* from the top surface layer for nanodimples and at a height *h* from the bottom for nanobumps. It is given by $f\left(t\right)=1-\frac{2\uppi (pt-{t}^{2})}{\sqrt{3}{p}^{2}}$ for our design of nanodimple metasurface. The refractive indices ${n}_{\text{pdms}}$ and ${n}_{\text{air}}$ are for PDMS and air medium with values of 1.45 and 1.00, respectively.

As the fill fraction varies with depth (or height), effective refractive index calculated using Eq. (6) also varies with depth or height. One may note that the fill fraction (and hence the effective refractive index) variation at different depth/height also depends on the pitch, shape, material of pattern surface and the surrounding medium. The gradient index profiles for ND-A1, ND-A2, NB-A3, ND-B1 and ND-B2 are shown in Fig. 4b. All the patterned surfaces will have a refractive index of 1.45 for the PDMS present below the pattern. But within the pattern, the refractive index varies as a function of depth/height. It is observed that with shallow patterns (such as the blue dotted line in Fig. 4b, the incident light encounters a higher refractive index (∼1.3) at the top surface of nanodimples and passes through a stiffer profile to reach 1.45. With a deep pattern (such as the green short-dash line), the refractive index at the top is lesser (∼ 1.1) and reaches the value of 1.45 only at a depth of 250 nm. It is observed that nanobump metasurface (NB-A3) of lower pattern height (∼ 72.1 nm) shows very low effective index at the top (∼ 1) and increases to ∼ 1.16 within the nanobump. Therefore, the response to the incident light will be different, and the extent of scattering will differ in each sample.

Similar to the measurement of diffuse intensity, total transmitted intensity is measured without and with the sample using the arrangement shown in Fig. 3b, and their ratio is plotted in Fig. 4c for the same set of samples. This ratio is defined in Eq. (4) earlier and does not exceed 1 in the ideal case. It appears to slightly exceed 1 for the planar samples for wavelength below 500 nm (as seen in the inset in Fig. 4c as they have very high transmission comparable to the measurement without the sample. Any minor fluctuations in source intensity or/and the background intensity can result in the ratio defined in Eq. (4) to go beyond unity. Some of the diffuse light from the closed Port-2 traveling backward can also increase the measured counts when the sample is present. The ratio representing total transmission is high (more than 0.9) for all the samples and without significant wavelength dependence as scattering has less effect on the spectral shape of total transmission spectrum but has significant effect on the total transmission intensity counts. The small differences between the samples (as seen in the inset) can be explained. To start with, glass and unpatterned PDMS have nearly equal total transmission and the maximum total transmission ratio among all the samples. ND-A2 (*d*_n_ = 0.340) and ND-B2 (*d*_n_ = 0.307) have nearly equal total transmission which is expected as both the samples have comparable *d*_n_ value. Nanobump metasurface (NB-A3) has total transmission lying in between that of ND-A1 with *d*_n_ = 0.179 and ND-A2 with *d*_n_ = 0.340, although nanobumps have *h*_n_ = 0.100 which is much less than the *d*_n_ value in nanodimples. An increase of texturing depth/height causes more scattering in transmission, reducing the total transmission and increasing the diffuse transmission. It follows a trend that is opposite of what was seen in diffuse transmission spectrum as a result.

Transmission haze is calculated using Eq. (5) with both the numerator and denominator measured in the presence of the sample. This is shown in Fig. 4d for all the samples along with their spectral variation. For planar glass and unpatterned PDMS, transmission haze is equal throughout the visible range, and has the least value. It is also observed that there is a greater transmission haze for ND-B2 with *d*_n_ = 0.307 compared to ND-B1 with *d*_n_ = 0.125. A similar trend where the transmission haze increases with increase of *d*_n_ is also observed for ND-A2 with *d*_n_ = 0.340 compared to ND-A1 with *d*_n_ = 0.179. We observe that ND-A2 (*d*_n_ = 0.340) and ND-B2 (*d*_n_ = 0.307) have comparable transmission haze. In addition, ND-A1 with (*d*_n_ = 0.179) and ND-B1 (*d*_n_ = 0.125) have lower and comparable transmission haze since the two samples have small and nearly equal *d*_n_ value. With an increase of *d*_n_ in patterned surfaces, scattering increases leading to more diffuse transmission in the estimation of total transmission. Transmission haze therefore increases with increase of the value of *d*_n_. In addition, transmission haze follows the same increasing trend with normalized depth as that of diffuse transmission. Nanobump metasurface (NB-A3) shows the highest transmission haze although it has the lowest pattern height compared to the pattern depths of the other structured surfaces and has intermediate value of both diffuse and total intensity counts ratio among the patterned surfaces. Thus, it is found that transmission haze is quite sensitive to the value of *d*_n_ (or *h*_n_) as well as to the shape of the pattern. All the measured samples have transmission haze decreasing with increase of wavelength from 400 to 550 nm, then remaining nearly constant up to 750 nm, and then increasing slowly at higher wavelengths.

As observed here, the metasurface with nanobumps has more transmission haze compared to a similar arrangement of nanodimples of comparable pitch. The reason is that the incident light encounters different refractive index profiles in nanodimples and nanobump surfaces. Here nanobumps (pitch of 716 nm) have an average height of 72.1 nm whereas the two samples of nanodimples of nearly equal feature spacing (715 and 730 nm) have an average depth of 128.0 and 248.6 nm. Together with their different feature characteristics (such as fill fraction and different depth/height), they provide a sharp difference in their graded refractive index profile for the incident light. When the incident light passes through different refractive index profiles in nanobumps and nanodimples, the incident light suffers different amount of scattering during its traversal through the samples. The variation of the effective refractive index profiles is shown in Fig. 4b and was discussed earlier. The nature of the spectrum indicates that the variations are due to the effect of sample as these do not resemble the shape of the source spectrum.

From the analysis of the above results, we see that diffuse transmission and transmission haze change with the value of *d*_n_. More specifically, both increase with increase in the value of *d*_n_. At the same time, the changes in the total transmission are not significant for most of the PDMS samples due to an increase in *d*_n_ value. This implies that these patterned samples can help in increasing the transmission haze due to their texturing without affecting total transmission to any significant extent. The spectra for total transmission and transmission haze being nearly flat over the wavelength range of 550 to 750 nm and transmission haze not exceeding 0.5 over the wavelength range of 500 to 800 nm for any of the samples makes it applicable to devices that work in green to red region of the visible spectrum. Further study to support the above observations on diffuse transmission or transmission haze is discussed in terms of wavelength-integrated far-field transmission profile in Section 4. Simulation results on total transmission and transmission haze are discussed in Section 5.

### Measurement and analysis of reflection haze

**Fig. 5:**
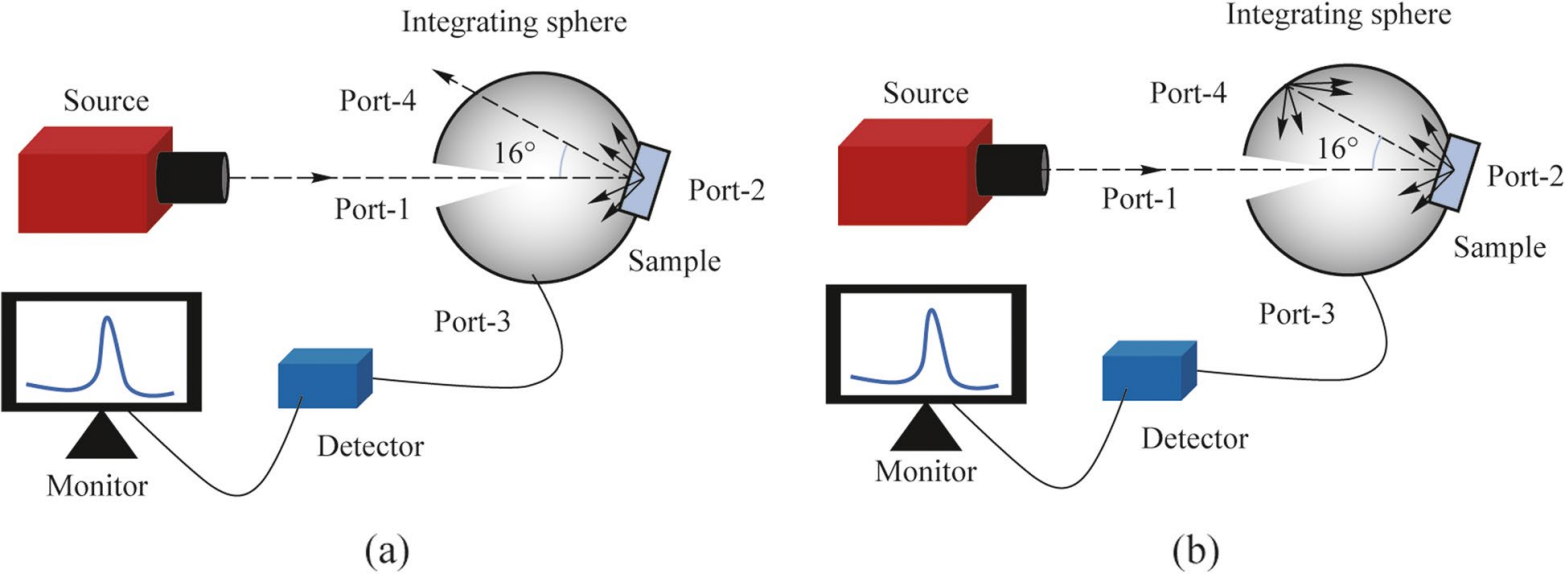
Schematic of the experimental arrangement to measure diffuse reflection and total reflection is shown in **a** and **b**, respectively

The arrangement to measure diffuse reflection and total reflection is shown in Fig. 5a and Fig. 5b, respectively. The source, detector and the integrating sphere used here are the same as in the transmission measurements, but with a different set of port combinations of the integrating sphere. The source is kept outside the integrating sphere and the collection fiber of the detector is kept at Port-3 as before. To remove the specular part during the diffuse reflection measurement, the sample is kept at an incidence angle of 8° just outside the sphere at Port-2, thus avoiding the contribution of transmitted light in reflection data. The contribution from specular reflection is removed by keeping Port-4 open, because the angle between Port-1 and Port-4 is 16°. On the other hand, the total reflection is measured by keeping Port-4 closed so that the specular reflection is also collected in the detector. The diffuse and total intensity counts in reflection are measured without any sample, and then individually with planar glass, unpatterned PDMS and patterned PDMS samples for the visible wavelength range of 400 – 800 nm. The ratios are calculated using Eqs. (3) and (4) and analyzed.

**Fig. 6:**
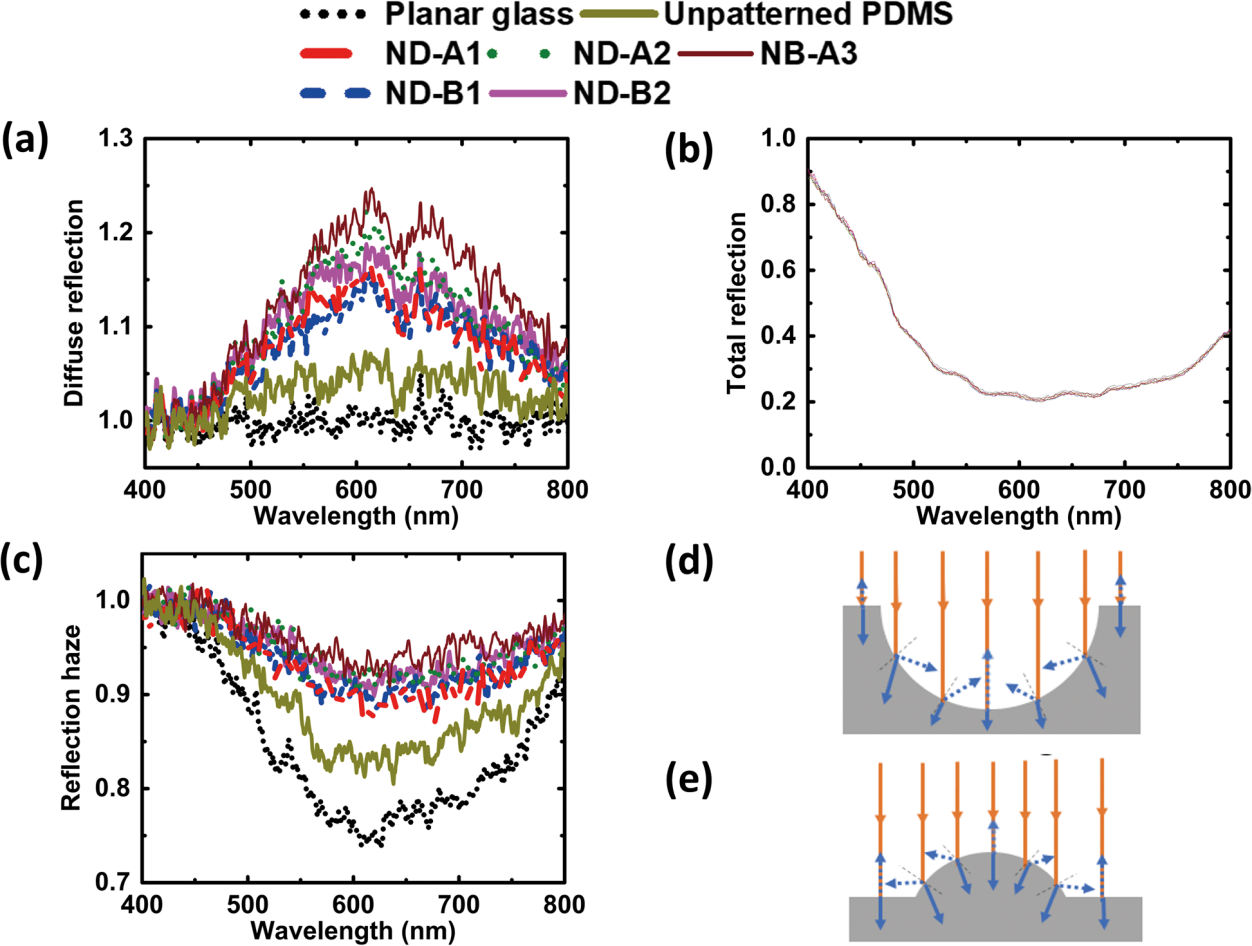
**a** Diffuse reflection. **b** Total reflection. The data of all the samples are coincident. **c** Reflection haze as a function of wavelength in the visible range. **d** and **e** represent the optical path variation of scattered light in nanodimple and nanobump metasurfaces respectively, for normally incident collimated light. The height of the bump in **e** is kept smaller than the depth of the dimple in **d** as the sample studied is of this type

Figure 6a represents the measured diffuse reflection data calculated using Eq. (3). These curves show a similar trend as seen earlier in diffuse transmission. The quantity goes beyond unity as it represents the ratio of diffuse intensity counts with and without the sample, and there is a higher diffuse scattering in the presence of the sample than in the absence of the sample. A wavelength-independent spectrum is observed for planar glass. Unpatterned PDMS also shows a similar spectrum but has slightly greater diffuse intensity counts ratio as compared to the planar glass due to the presence of the glass below the PDMS layer during these measurements. In the patterned samples, the diffuse intensity counts increase with the increase of normalized depth *d*_n_ and also have a wavelength dependence similar to the case of diffuse transmission discussed earlier. For nanodimple surfaces, the maximum diffuse reflectance is observed for ND-A2 with *d*_n_ = 0.340. It is noted that ND-A1 with *d*_n_ = 0.179 has lesser diffuse reflection, although both the samples have comparable value of pitch (*p*). Similar trend is also observed for ND-B2 with *d*_n_ = 0.307 and ND-B1 with *d*_n_ = 0.125. In addition, ND-B1 with *d*_n_ = 0.125 has slightly lower intensity counts ratio compared to ND-A1 with *d*_n_ = 0.179 due to its slightly smaller value of *d*_n_. Nanobump metasurface (NB-A3) with *p* = 716 nm and *h*_n_ = 0.100 has the highest diffuse reflection compared to all the nanodimple structures and planar structures studied in this section. Dimple structure has a steeper graded index profile compared to bump structure due to the different shape profile and as a result nanobump has higher diffuse reflection. For all the structured surfaces of PDMS, diffuse reflection is larger than the unpatterned PDMS in the wavelength range of 500 to 800 nm. This could be due to the chosen value of pitch (561, 566, 715, 716, and 730 nm) being comparable to this wavelength range and thus producing more diffuse scattering in that range. It is interesting to note that the values of diffuse reflection lie between 1 and 1.25 for all the samples at all the measured wavelengths. In comparison, the diffuse transmission discussed earlier varied over a range six times wider from 1 to 2.5. This is attributed to the transparent nature of these samples with high transmission and low specular reflection.

Figure 6b represents the measured total reflection obtained from Eq. (4). The total reflection for both structured and planar surfaces is lowered with increase of wavelength from 400 nm, and reaches a flat minimum at around 550 nm. It starts increasing again after 750 nm. All the samples have very similar values of total reflection for all the wavelengths in the range of 400 to 800 nm, to the extent that the different plots are indistinguishable in Fig. 6b. The pitch and pattern depth appear to have no effect on total reflection. The minimum value of total reflection from all the samples is quite low at 0.2. A decrease of specular reflection with increase of normalized depth [30] may get compounded with an increase in diffuse reflection, making the sum of specular and diffuse reflection i.e. total reflection to be highly comparable for all the samples.

Figure 6c depicts the reflection haze calculated using Eq. (5) from the above reflection data. Planar glass has the lowest reflection haze. Unpatterned PDMS has more reflection haze than the planar glass. The patterned surfaces have higher reflection haze than the planar samples. It is observed that reflection haze follows the same trend with normalized depth as the diffuse reflection. Reflection haze is lesser for lower values of normalized depth *d*_n_. Nanodimple surface of ND-A2 with *d*_n_ = 0.340 shows higher reflection haze than ND-A1 with *d*_n_ = 0.179. A similar trend is observed for two samples ND-B2 and ND-B1 with comparable pitch (561 and 566 nm) but having different normalized depths of 0.307 and 0.125. For reflection haze too, PDMS with triangular arrangement of nanobumps (pitch of 716 nm) has higher values than PDMS with nanodimples of comparable pitch. The shape of the pattern and its depth / height makes a significant contribution to the reflection haze as in the case of transmission haze.

It is noteworthy that in planar glass and unpatterned PDMS on glass, the values of transmission haze were comparable. But they are distinctly different for reflection haze. Unpatterned PDMS on glass has more reflection haze than planar glass. This indicates that the reflection haze is highly dependent on the surface roughness, even in the absence of intentionally-made periodic patterns. This haze gets compounded in the presence of patterns on PDMS and increases further. The fluctuations in measurement are also more in reflection mode. In addition, the wavelength dependence in 400 to 800 nm is significantly reduced for the reflection haze in patterned samples. The nanobump surface has the largest reflection haze among all the patterned samples, similar to the case of transmission haze. The magnitude of reflection haze is 0.9 or more for the patterned samples (Fig. 6c), while the transmission haze for the same set of samples was lesser, lying between 0.2 and 0.4 in Fig. 4d. The reflection haze in Fig. 6c slightly exceeds unity for wavelengths below 500 nm for some samples. Reflection haze is the ratio given in Eq. (5) for each sample. The magnitude of these quantities is comparable for wavelengths below 500 nm for all the samples. Therefore, any minor fluctuation in measured intensity will lead to the ratio exceeding unity.

The pictorial representation of the optical path variation in the two shapes of dimples and bumps is shown in Fig. 6d and Fig. 6e, respectively. When the light is incident normal to the patterned PDMS layer, only a small portion of the incident light is reflected back as specular reflection. Most of the incident light suffers oblique incidence due to curved shape of dimple and bump structures and gets reflected back as diffuse light from the curved surface. Some part of the incident light may also undergo multiple scattering/reflection from the curved surface before it gets detected as diffuse reflected light. If normalized depth/height increases, the curved part of the pattern on the surface increases. This makes more of the incident light to experience oblique incidence at the pattern, even though it is incident normal to the bulk PDMS layer. This leads to generation of more diffused light with increase of normalized depth/height. Also, it is to be noted that chances of multiple scattering/reflection are more in nanodimples than nanobumps due to their larger depths used here. This implies that there could be greater optical path for diffused reflected light in nanodimple than in nanobump structures, indicating that with increase of normalized depth/height, optical path increases for diffuse reflected light. The patterned surfaces with larger depth, namely ND-B2 with *d*_n_ = 0.307 and ND-A2 with *d*_n_ = 0.340 demonstrate that diffuse reflection increases with increase of the normalized depth *d*_n_. The reason as discussed before is that with increase of *d*_n_, the shape of the features introduces more spatial modulation on the layer. As a result, most of the collimated light beam incident normal to the surface experiences oblique incidence inside the pattern as seen in Fig. 6d, e. This leads to reflected light going in directions other than the expected specular direction, and thus increasing the diffuse component in total reflected intensity as well as the reflection haze.

It is also observed that for comparable pitch of 730 and 716 nm, PDMS layer with nanodimples (depth 248.6 nm) has lesser diffuse reflection than PDMS with nanobumps (height 72.1 nm). The reflected light has a greater set of angles for optical path in nanodimples than in nanobumps as seen in Fig. 6d, e, which in turn produces the effect of capturing more reflected/scattered light for nanodimples in the diffuse light measurement than in nanobumps. The difference in the depth profile of effective refractive index of the patterned surface for the cases of nanodimples and nanobumps also leads to different magnitudes of diffuse spectra. These two effects lead to production of more scattered waves in reflection in nanobumps than nanodimples. For all the structured surfaces of PDMS, diffuse reflection is larger than the unpatterned PDMS in the wavelength range of 500 to 800 nm. This could be due to the chosen value of pitch (561, 566, 715, 716, and 730 nm) being comparable to this wavelength range and thus producing more diffuse scattering in that range. From the analysis of above results, we see that reflection haze increases with increase in the value of *d*_n_ even though there is no observable change in the total reflection for the structured and unpatterned PDMS samples. For the wavelength range studied here, nanodimple surfaces with larger normalized depth_,_ and nanobump surface of even smaller normalized height have higher diffuse reflection and higher reflection haze.

From our results, it is observed that transmission haze can be practically doubled from a value below 0.2 (for planar surface) to a value close to 0.4 (using patterned surfaces) in a predictable manner in the visible range, as the haze increases with the increase of normalized depth *d*_n_ in nanodimple surfaces. In addition, nanobump surface with a specific height of the bump can result in much larger transmission haze compared to nanodimple surface with an equivalent value for the depth of the dimple, for comparable pitch. These are relevant to pitch sizes lying in the range of 550 to 760 nm in these measurements. Reflection haze also shows a similar trend with increase of *d*_n_, but the values of reflection haze are much larger (exceeding 0.9) irrespective of pattern depth and pattern shape of dimple and bump. One may note that reflection haze is more than 0.75 for planar glass and more than 0.80 for unpatterned PDMS in the visible range. Therefore, the option to increase it in smaller steps with the help of patterning parameters (such as height or depth) is minimal.

## Measurement and analysis of wavelength integrated far-field transmission profile

**Fig. 7:**
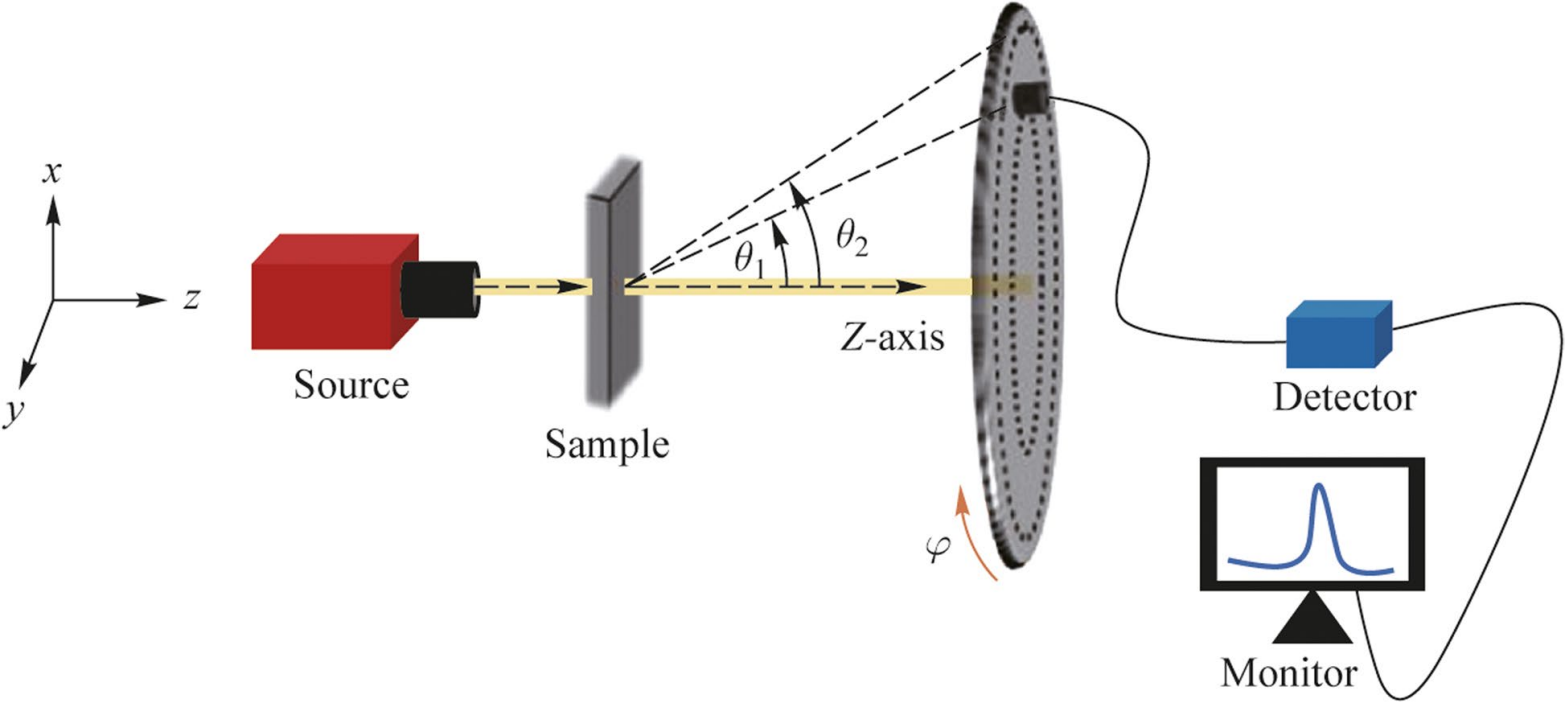
Experimental arrangement to measure wavelength-integrated far-field transmission profile of samples

While the pitch values of the patterned samples are 561, 566, 715, 716, and 730 nm, the wavelength range studied here spans over 400 to 800 nm. Therefore, it is likely that a certain amount of light can be diffracted in the lower range of wavelength. This can be ascertained by studying the far-field transmission profile. In addition, the effect of variation in wavelength can be discounted by analyzing the wavelength-integrated values so that all the samples can be compared in their performance. To find the scattering effect from the patterned and planar samples, study of wavelength-integrated far-field transmission profile $\sigma$(*θ, φ*) is explored. This is defined as [2]

7 $$\sigma \left(\theta , \varphi \right)=\frac{\sum_{i}{I}_{i}(\theta , \varphi ) {\lambda }_{i}}{\sum_{i}{\lambda }_{i}},$$

where* I*_*i*_ is the transmission intensity count corresponding to a specific wavelength *λ*_*i*_. The angles *θ* and *φ* are the polar angle and azimuthal angle respectively with reference to the incident direction of light as shown in Fig. 7. The quantity in Eq. (7) is the expectation value of intensity counts over a spectral range or normalized area (in counts) under a spectrum. This quantity is represented by $\sigma .$ The summation spans the wavelength range of 400 to 800 nm in this study. The experimental setup to measure the wavelength-integrated far-field transmission profile is shown in Fig. 7. This consists of a white light source (Thorlabs, SLS201L). The sample is present in the *xy* plane at a distance of 5 cm from the source and a home-made detector mount is kept in the *xy* plane at a distance of 21.5 cm from the sample. The wavelength range of measurement is 400 to 800 nm and the step size of wavelength variation is 0.5 nm. All the measurements are done in free space without any confinement of transmitted light. A fiber-coupled detector connected to a spectrometer (StellarNet, detector module for visible range) can be positioned at different values of *θ* and *φ* and used to collect the transmitted intensity. The numerical aperture of the fiber ferrule decides the cone angle of light collection. The home-made detector mount helps in collecting the transmitted light along both azimuthal angle (*φ*) and polar angle (*θ*) variations as shown in Fig. 7. Both these angles can be changed only in a step size of 1°. For the measurements, *φ* is varied from 0 to 360° with step size of 10°, while *θ* values of 0, 5° and 8° were sufficient for the analysis. Due to the instrumental constraint, very small *θ* values of 1° to 4° could not be measured. As no variation in intensity was observed at different *φ* for *θ* > 8°, larger angles were not tested.

**Fig. 8:**
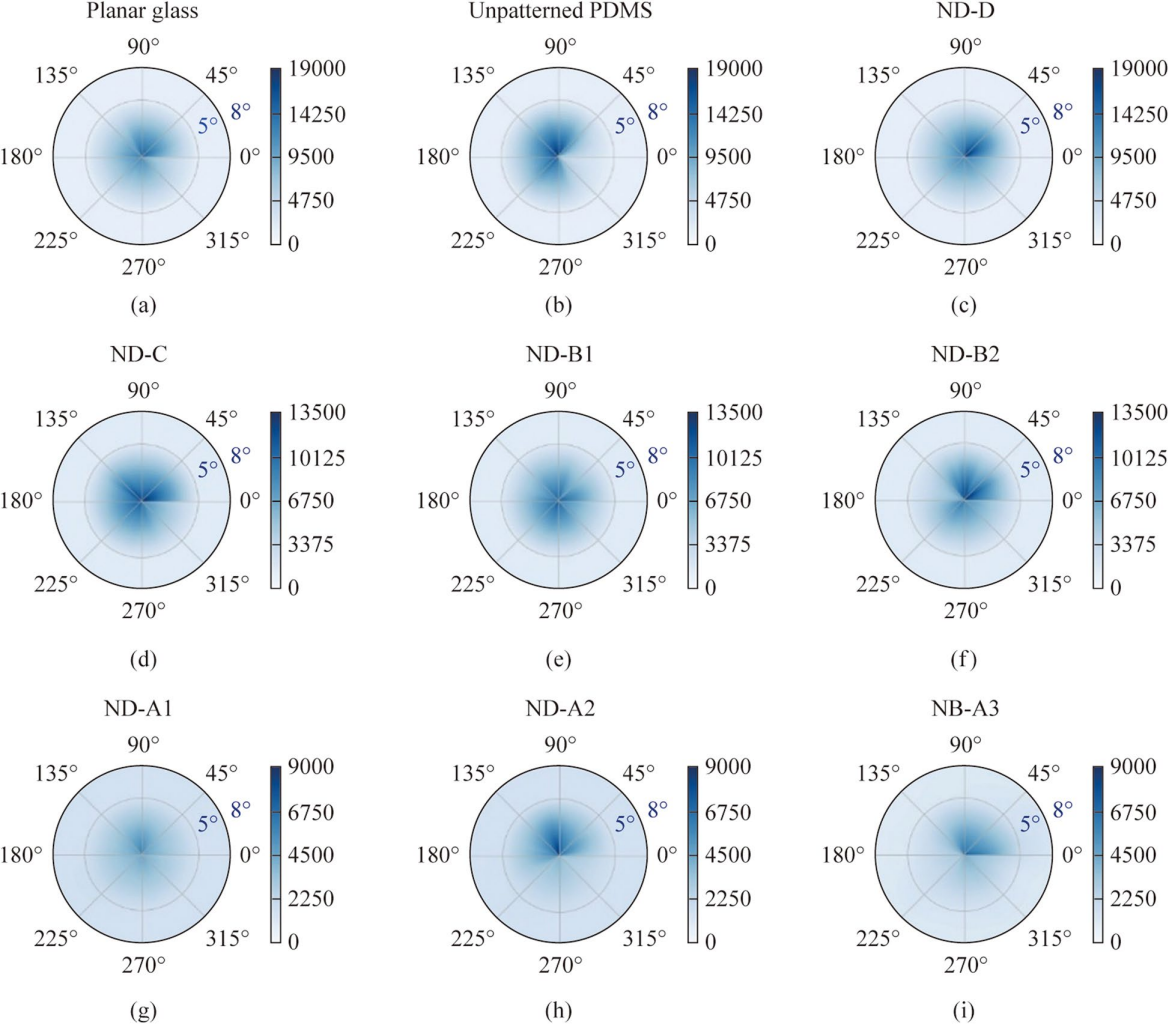
Wavelength-integrated far field transmission profile shown for different samples for polar angle range of 0^o^ < *θ* < 8° and azimuthal angle range of 0° < *φ* < 360°. **a**, **b**, **c** Planar glass, unpatterned PDMS and ND-D, **d**, **e**, **f** ND-C, ND-B1 and ND-B2, **g**, **h**, **i** ND-A1, ND-A2, NB-A3, respectively. These use the data of $\sigma$ interpolated for intermediate angles. The color bar represents the value of $\sigma$ calculated over the spectral range of 400 to 800 nm

The results of wavelength-integrated far-field transmission profiles for different planar and structured surfaces are shown in Fig. 8. These are contour plots with the color bar representing the value of $\sigma$ calculated over the spectral range of 400 to 800 nm. For the continuous and gradient variation respectively along the azimuthal and polar angles, the measured data are interpolated using linear interpolation. The plots in Fig. 8a, b correspond to the cases of planar glass and unpatterned PDMS respectively. Neither shows a variation in $\sigma$ for *θ* > 2°. The profile is not a perfect circle. This anisotropy in *φ* is present even in the absence of the sample. The effect of numerical aperture of the fiber ferrule (detector) and the shape of the light beam will also have some influence on the measurements. In Fig. 8c, d, the wavelength-integrated far-field transmission profiles are shown for nanodimple metasurfaces of extremely small pattern depths, namely ND-D (depth: 7 nm) and ND-C (depth: 14 nm). These samples also have smaller pitch values of 198 and 255 nm, which are lower than the values studied in the earlier sections and depth values of 7 and 14 nm which are also among the least available with us. The trends are comparable to those seen for the planar samples.

In Fig. 8e, f, the profiles are shown for ND-B1 (depth: 71.0 nm) and ND-B2 (depth: 172.7 nm), while in Fig. 8g, h, i, the profiles are shown for ND-A1 (depth: 128 nm), ND-A2 (depth: 248.6 nm) and NB-A3 (height: 72.1 nm) respectively. The plots imply that transmission intensity counts along *θ* = 0 decrease with increase of both pitch and depth as the light scatters to higher angles. It is also observed that light scatters more to higher angles for higher dimensional samples. To show this increase from ND-A1 to ND-A2 as well as from ND-B1 to ND-B2 more clearly, the maximum value of $\sigma$ (range of color bar) has been changed for the three rows of pictures in Fig. 8. Samples ND-A1, ND-A2 and NB-A3 show similar type of transmission scattering behavior. ND-C, ND-B1 and ND-B2 have lower transmission scattering angle of light compared to higher pitch samples and these three samples show similar type of behavior in scattering. Planar glass, unpatterned PDMS and ND-D have insignificant transmission scattering effect. This implies that pitch has more significant effect than depth on the wavelength-integrated far-field transmission measurements because the range of the wavelength used here is 400 to 800 nm.

From these plots, it is observed that for patterned surfaces with increasing pitch and increasing depth, transmitted light scatters to slightly higher value of *θ*. Moreover, it is observed that scattering in transmission increases with increase of normalized depth *d*_n_, except ND-B2 with depth 172.7 nm where it shows a decreasing trend in transmission scattering. Thus, these plots indicate that with both increasing pitch and depth of the features, scattering in transmission mode is increased. Overall if we compare all the samples used, there is a clear trend that with increase of *d*_n_, scattering increases and light scatters to higher values of polar angle (*θ*). In transmission, there is a similarity between wavelength-integrated far-field transmission profiles and the diffuse transmission or transmission haze. The scattering being confined to smaller values of *θ* in transmission is explained by the pictorial representation in Fig. 6d, e where the rays passing through PDMS lie in a small cone. Results are shown here only for transmission because far-field reflection data in free space in the absence of an integrating sphere is not reliable. Wavelength integrated transmission profiles support the increase in scattering, i.e., increase in diffuse transmission with increase of the normalized depth *d*_n_ of the dimples in transmission.

## Simulation results and discussion

Some of the earlier work [40, 41] on simulation of haze has shown that haze is fully dependent on the size, shape, depth/height of the patterns and the material aspects of the patterns and the surface. It was also seen that there is a nearly linear relationship between the parameters of the patterned surface and optical haze generated by the patterns on the surface. A similar trend is also seen in our experimental results, where both transmission haze and reflection haze were seen to increase with increase of the normalized depth for nanodimple metasurfaces, and even when the curvature of the pattern on the metasurface was changed from concave to convex in nanobump metasurface.

We have performed simulations for transmission haze based on the finite element method (FEM) using the 3D version of wave optics module of the commercially available COMSOL Multiphysics software version 6. Transmission haze is calculated for the structures of ND-A1, ND-A2, and NB-A3 metasurfaces for comparison with the experimental results presented earlier. ND-A1 and ND-A2 have nanodimples arranged in a triangular lattice while NB-A3 has nanobumps placed in a similar fashion on PDMS substrate. To be comparable to the experimental conditions, we considered that the metasurfaces are placed on glass substrate, even in the simulations. The refractive index for the PDMS and glass are taken as 1.45 and 1.50 respectively, and the other simulation parameters for these samples are taken from Table 1. The computations were carried out for light in the form of plane waves incident normally (along the *z*-axis) on the metasurfaces present in the *xy* plane. The wavelength range considered for the simulation is from 400 to 800 nm, with 1 nm interval. The triangular lattice structure in our samples implies that the unit cell will be rectangular.

**Fig. 9:**
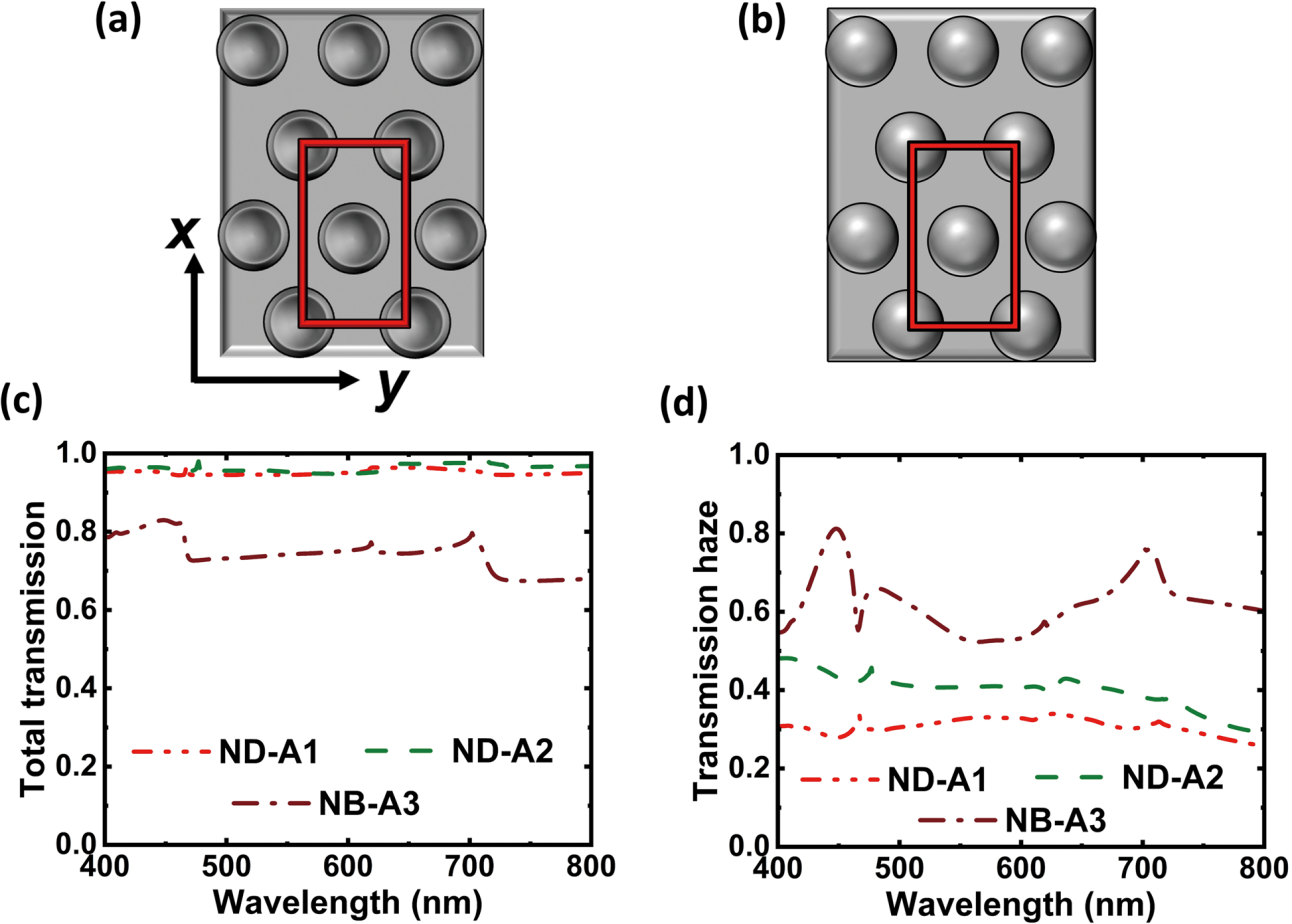
Simulation results. Schematic of the **a** nanodimple and **b** nanobump metasurface in the *xy* plane are shown; the red rectangle in them represents the unit cell of the calculation. The calculated values of total transmission and transmission haze are shown in **c** and **d** respectively for ND-A1, ND-A2 and NB-A3, at a fixed polarization of ${E}_{x}=0.75, {E}_{y}=0.25$

Figures 9a, b show the schematic of the nanodimple and nanobump metasurface respectively in the *xy* plane. The unit cell considered in our calculation is shown by the red rectangle. Periodic boundary condition is applied in *x* and *y* directions and the perfectly matched layer is applied in the *z* direction to truncate the computational domain. Scattering boundary condition is applied at the top and bottom surfaces of the structure to reduce the unwanted reflections. Total transmission and specular transmission were calculated in the chosen wavelength range for the metasurfaces, and their difference gives the diffuse transmission. The transmission haze is obtained as the ratio of diffuse transmission with the total transmission [41]. It is important to note that the metasurfaces are polarization sensitive. Transverse electric (TE) and transverse magnetic (TM) polarizations correspond to the electric field (E) of incident light present along the *x*-axis and *y*-axis respectively.

In the experiments described in the earlier sections, we used unpolarized light incident normally on the sample along with in-plane arbitrary orientation of the sample, to be compatible with standard applications of optoelectronic devices. Therefore, the simulations for total and specular transmission are also performed for arbitrary combinations of electric field magnitudes (*E*_*x*_ and *E*_*y*_) along the reference *x* and *y* axes of the sample. When the contribution of the electric field along the two axes is equal, the highest transmission haze is obtained for ND-A1 and ND-A2. Subsequently, we calculated the transmission haze for several mixtures of the magnitudes of *E*_*x*_ and *E*_*y*_. Figures 9c, d depict the calculated values of total transmission and transmission haze respectively for one case of ${E}_{x}=0.75, {E}_{y}=0.25$ from ND-A1, ND-A2 and NB-A3 metasurfaces. It is seen that the total transmission (Fig. 9c) is comparable for the two nanodimple metasurfaces of ND-A1 and ND-A2, while it is lesser for the nanobump metasurface (NB-A3). In Fig. 9d, ND-A1 has the lowest transmission haze (∼0.3), which increases for ND-A2 (∼0.4) and it further increases for NB-A3 metasurface (∼0.6). Thus, NB-A3 shows the highest transmission haze among the three cases studied here. In comparison, the transmission haze obtained in our measurements (Fig. 4d) in the wavelength range of 500 to 700 nm were approximately 0.2, 0.3 and 0.4 respectively for ND-A1, ND-A2 and NB-A3. As the orientation of the electric field of incident light during the measurement was not kept fixed with reference to the *x* and *y* axes of the sample (Fig. 9a, b), such a reduction in the experiment can be accounted for. Besides, the over-all trends are found to be comparable between the simulations and the measurements, since lower transmission haze is obtained for ND-A1 due to its lesser *d*_n_ and higher transmission haze is obtained for NB-A3 due to its pattern shape in spite of having the lowest *h*_n_.

## Conclusion

Colloidal crystals were grown by self-assembly from monodispersed colloids of different diameters. These were used as master mold to fabricate patterned PDMS layers using easy and cost-effective soft imprint lithography technique. The measurements of diffuse and total transmission obtained as a function of wavelength with and without the samples show that diffuse transmission and transmission haze can be increased by increasing the ratio between the depth of the feature and pitch of the feature, without affecting the total transmission. With an increase of normalized depth *d*_n_ from 0.169 to 0.328, the haze increases on average from 0.2 to 0.4. With increase in depth, the size of the pattern is also increased, even though the pitch is unchanged. Thus, by controlling the normalized depth *d*_n_, we can increase or decrease the diffuse transmission/haze without affecting the total transmission. It is also observed that there is an increase of reflection haze with the increase of normalized depth but the variation of total reflection with normalized depth is negligible. Apart from size and depth of dimple patterns, it is also possible to change the shape and make patterns with bumps. These give transmission and reflection haze which are more than those obtained with nanodimple surfaces. Both the nanodimple and nanobump metasurfaces give wavelength-independent transmission and reflection haze in the range of 550 to 750 nm. The wavelength-integrated far-field transmission profiles measured on all the samples confirm that the scattering in transmission direction is restricted to smaller angles. Simulation results on nanodimple and nanobump metasurfaces with comparable pitch establish that the nanobump metasurface gives higher transmission haze even when its pattern height is lesser than the pattern depth of the nanodimple metasurface. Thus, the three parameters of pattern size, shape and depth/height are sufficient to control reflection haze or transmission haze without affecting total reflection or total transmission, which in turn increases the options for different optoelectronic applications to improve the efficiency of devices.

## Data Availability

The data that supports the findings of this study are available from the corresponding author, upon reasonable request.
