# Correction: Control of visible-range transmission and reflection haze by varying pattern size, shape and depth in flexible metasurfaces

**DOI:** 10.1007/s12200-024-00135-1

**Published:** 2024-08-25

**Authors:** Avijit Maity, Vaswati Biswas, R. Vijaya

**Affiliations:** 1 Department of Physics, Indian Institute of Technology Kanpur, Kanpur, 208016 India; 2 Centre for Lasers and Photonics, Indian Institute of Technology Kanpur, Kanpur, 208016 India

**Correction: Frontiers of Optoelectronics (2024) 17:25** 10.1007/s12200-024-00125-3

Following publication of the original article [1], the author found an error in Fig. 4(b), which has been updated from the earlier figure:
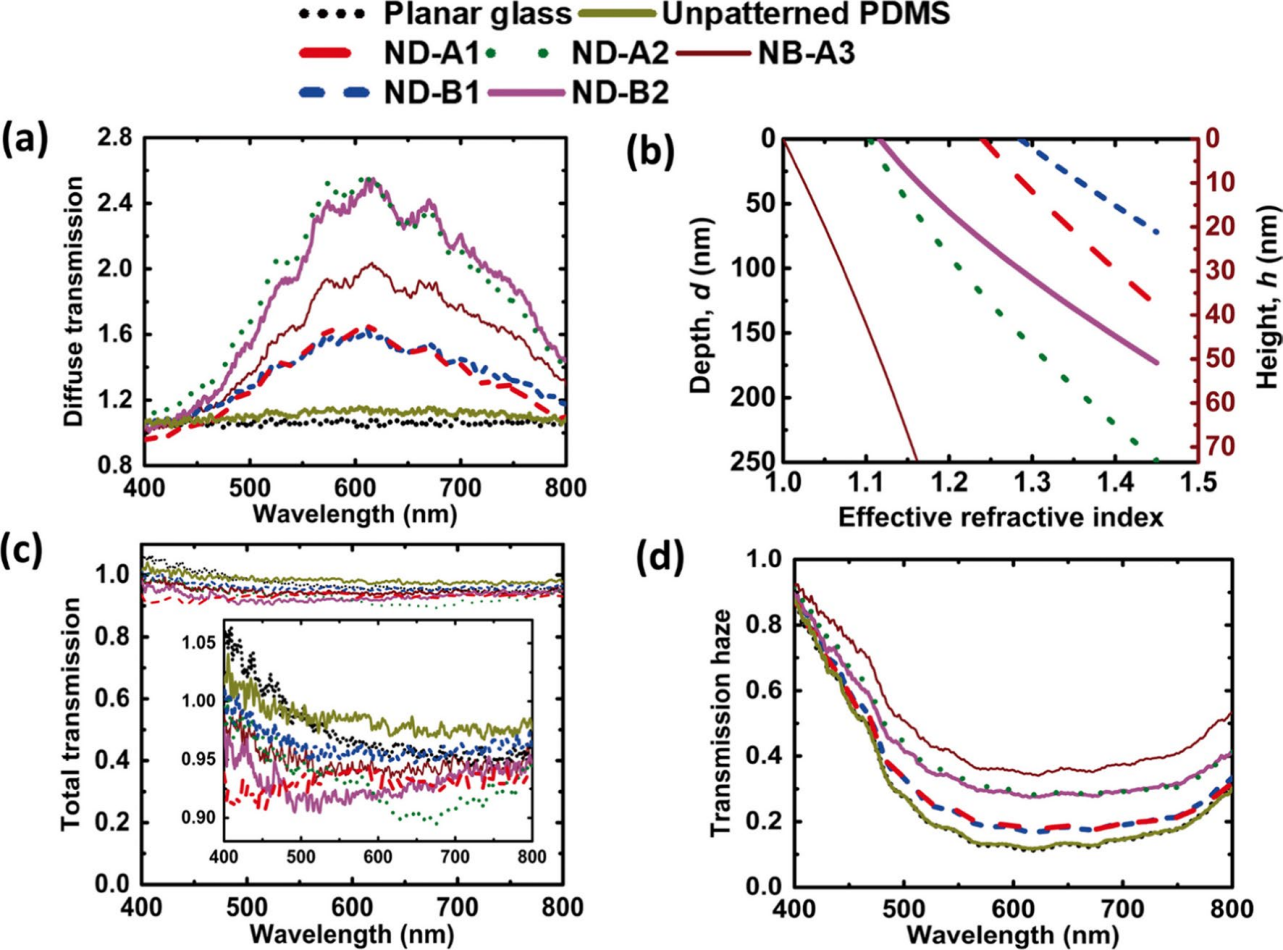


To the corrected figure:
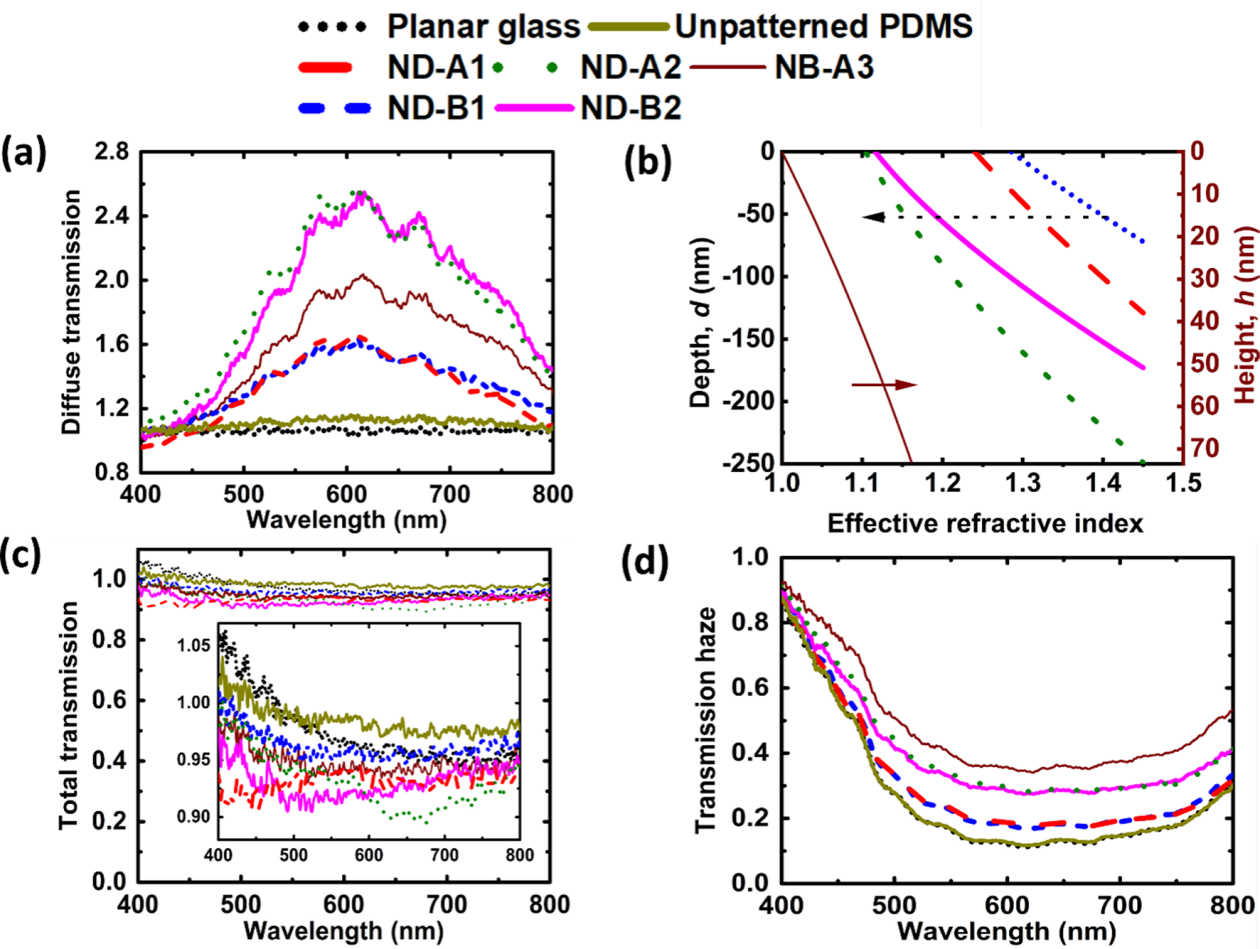


The original article [1] has been updated.
